# Review on the Scale-Up Methods for the Preparation of Solid Lipid Nanoparticles

**DOI:** 10.3390/pharmaceutics14091886

**Published:** 2022-09-06

**Authors:** Sakshi V. Khairnar, Pritha Pagare, Aditya Thakre, Aswathy Rajeevan Nambiar, Vijayabhaskarreddy Junnuthula, Manju Cheripelil Abraham, Praveen Kolimi, Dinesh Nyavanandi, Sathish Dyawanapelly

**Affiliations:** 1 Department of Pharmaceutical Science and Technology, Institute of Chemical Technology, Mumbai 400019, India; 2 Department of Pharmaceutical Sciences, University of Nebraska Medical Center, Omaha, NE 68198, USA; 3 Department of Pharmaceutics, UCL School of Pharmacy, University College London, 29-39 Brunswick Square, London WC1N 1AX, UK; 4 Independent Researcher, 4/2 Moore Street, Gwynneville, Wollongong, NSW 2500, Australia; 5 Drug Research Program, Faculty of Pharmacy, University of Helsinki, Viikinkaari 5 E, 00790 Helsinki, Finland; 6 Department of Engineering Mathematics, University of Bristol, Biomedical Sciences Building, University Walk, Bristol BS8 1TD, UK; 7 Department of Pharmaceutics and Drug Delivery, School of Pharmacy, The University of Mississippi, Oxford, MS 38677, USA; 8 Pharmaceutical Development Services, Thermo Fisher Scientific, Cincinnati, OH 45237, USA

**Keywords:** solid lipid nanoparticles, scale-up, drug delivery, nanomedicines, high-pressure homogenization

## Abstract

Solid lipid nanoparticles (SLNs) are an alternate carrier system to liposomes, polymeric nanoparticles, and inorganic carriers. SLNs have attracted increasing attention in recent years for delivering drugs, nucleic acids, proteins, peptides, nutraceuticals, and cosmetics. These nanocarriers have attracted industrial attention due to their ease of preparation, physicochemical stability, and scalability. These characteristics make SLNs attractive for manufacture on a large scale. Currently, several products with SLNs are in clinical trials, and there is a high possibility that SLN carriers will quickly increase their presence in the market. A large-scale manufacturing unit is required for commercial applications to prepare enough formulations for clinical studies. Furthermore, continuous processing is becoming more popular in the pharmaceutical sector to reduce product batch-to-batch differences. This review paper discusses some conventional methods and the rationale for large-scale production. It further covers recent progress in scale-up methods for the synthesis of SLNs, including high-pressure homogenization (HPH), hot melt extrusion coupled with HPH, microchannels, nanoprecipitation using static mixers, and microemulsion-based methods. These scale-up technologies enable the possibility of commercialization of SLNs. Furthermore, ongoing studies indicate that these technologies will eventually reach the pharmaceutical market.

## 1. Introduction

**Figure 1:**
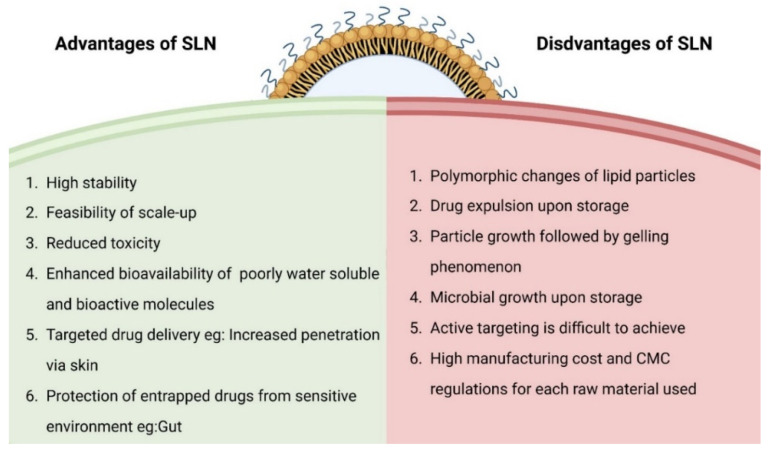
Advantages and disadvantages of SLN, data summarized from references [7,8].

Solid lipid nanoparticles (SLNs) were designed in the early 1990s as an alternative nanomedicine to other lipid-based carriers [1,2,3,4,5]. SLN dispersions are identical to oil in water emulsions, but the liquid lipid of the emulsion is replaced by a solid lipid (at room temperature), which can hold both hydrophilic and hydrophobic drugs [6]. SLNs offer distinctive advantages such as protecting active pharmaceutical ingredients, enhanced bioavailability, and increased penetration upon topical application [7,8]. There are several cosmetic products available on the market based on these attributes [9]. At the same time, SLNs face challenges such as polymorphic changes of lipid components, drug leakage and microbial growth upon storage. Despite several products being available on the cosmetics side, there are hardly any clinical trials active in the clinical trial database for other applications. One of the main reasons we assume for this is that the cosmetic regulations are often not as stringent as drug products. The advantages and disadvantages of SLNs are summarized in Figure 1.

### Rationale for Large-Scale Production

To enter the pharmaceutical market, specific requirements have to be met, i.e., having affordable, large-scale production techniques along with concurrent compliance with regulatory standards [10]. An easy production process running in a laboratory setup becomes useful only when it can be taken to a large scale. Large-scale processes require qualified lines to function effectively. The regulatory authorities qualify and accept high-pressure homogenization (HPH) lines to be used for parenteral nutrition [11].

The efficiency of SLNs is significantly determined by the process of synthesis, which in turn governs the size of the particle, drug loading capacity, release of drugs, drug stability, etc. Various methods are available for developing finely distributed lipid nanoparticle dispersions. Some manufacturing techniques, such as HPH and microemulsion dilution, have shown scale-up potential, a necessary condition for the launching of a product to the market [12]. Pharmaceutical firms require quick-to-implement, flexible (in terms of formulation type and sterility requirement), and low maintenance (in terms of cleaning) manufacturing lines to generate nanoparticles for clinical testing and commercialization. Converting a production procedure from a lab setting to a larger one is a difficult process, and if it is not performed accurately, the quality of the final product will differ. Undoubtedly, suitable modifications of manufacturing lines to handle scalable formulations and examine vital process parameters are crucial for evaluating the quality of the finished product [13].

The unavailability of a large-scale production process that produces a product of acceptable grade for regulatory agencies (US FDA) has hampered the commercialization of SLNs. Fundamental technical issues (for example, basic scale-up problems, toxicologically harmful byproducts from the production procedure) and regulatory elements such as the suitability of the manufacturing facility and production method to be approved and verified are the prevailing reasons behind this deficiency [4]. A large-scale production facility is required for commercial applications to bring out enough formulations for clinical trials. Furthermore, continuous processing is becoming more popular in the pharmaceutical sector to reduce product batch-to-batch differences [14].

The progress on conventional methods, characterization, and applications is reviewed in published reports [15,16,17]. Paliwal et al. reviewed recent research publications and patents of SLNs. In addition, authors discussed the controlled and targeted drug delivery applications of SLNs [15]. A recent review by Akbari et al. focused on manufacturing methods of SLNs, routes of administration and their applications in gene and peptide delivery systems. Moreover, the review covered regulatory status, commercialization plan and safety of SLNs [16]. Duan et al. reviewed physicochemical characterization, materials and methods used for the production of SLNs. Furthermore, challenges of stability, storage conditions and their applications are also well documented [17]. Researchers explored SLNs for therapeutic indications delivered using different routes of administration [16,18]. However, there is a need for information specific to the scale-up methods of SLNs.

**Figure 2:**
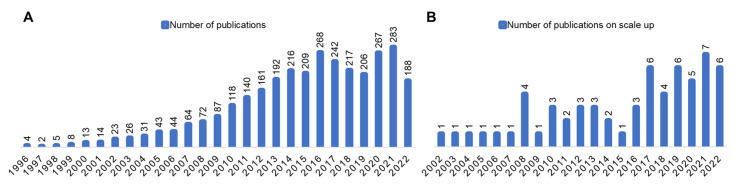
Bibliographic search analysis of SLNs from the PubMed database [19]. Search term “Solid lipid nanoparticles [Title/Abstract]” (**A**) yielded 3143 publications; “Scale-up of solid lipid nanoparticles [Title/Abstract]” yielded 62 publications (**B**).

The present review focuses on the rationale for large-scale production and further covers recent progress in scale-up methods for the synthesis of SLNs, including high-pressure homogenization (HPH), hot melt extrusion coupled with HPH, microchannels, nanoprecipitation using static mixers, and microemulsion-based methods. Our bibliographic analysis from the PubMed database (date 1 August 2022) revealed that the number of publications reporting (see Figure 2) the scale-up methods is only a fraction of articles reporting exploratory studies or other novel findings, implying the need for additional efforts.

## 2. Solid Lipid Nanoparticle Preparation Techniques

Generally, the SLN preparation techniques yield either dispersion (Section 2.1, Section 2.2, Section 2.3, Section 2.4 and Section 2.5) or solid powder forms (Section 2.6 and Section 2.7), which are discussed in the following subsections. Specifically, the SCF method and spray-drying method generate a solid form. Furthermore, any dispersion can be converted into solid form by using an optimized freeze/spray drying technique.

### 2.1. Ultrasonication

Ultrasonication is a dispersing technique that was first used to create stable lipid nanodispersions. Ultrasonication operates by dispersing molten lipids into minute droplets in a continuous phase. This method creates SLNs without using organic solvents and is fast, simple, and efficient. However, it has the disadvantage of necessitating an additional filtration step of the formulated SLN emulsion to eliminate impurities such as metal generated by ultrasonication, and it is frequently hindered by the occurrence of microparticles [5]. The idea behind this technique is to use sound waves to reduce particle size [17].

**Figure 3:**
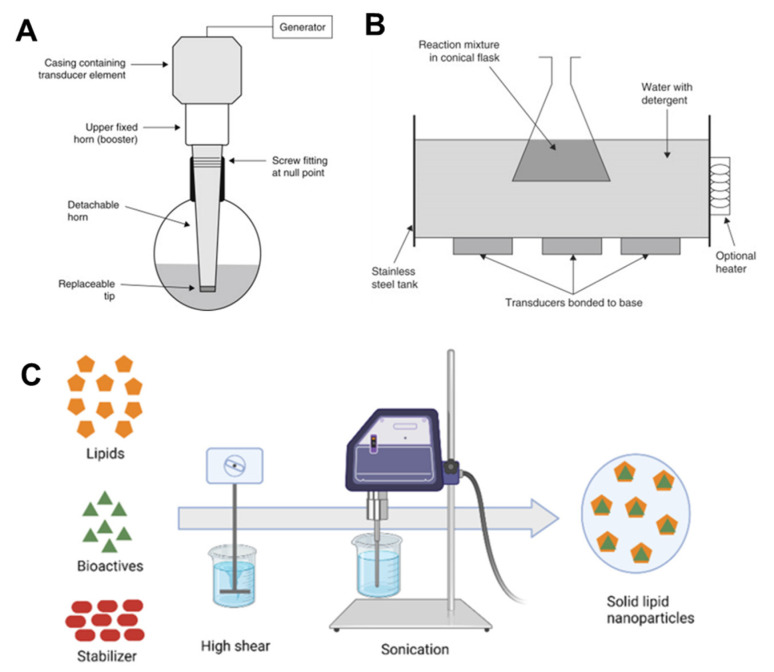
Schematic representation of the sonication method for the SLN probe sonicator (**A**), bath sonicator used in academic labs (**B**) and SLN preparation by the sonication method (**C**). In this technique, the lipids will be melted and the aqueous phase with surfactants is then sonicated using probe sonicator to form emulsions with reduced droplet size. Gradual cooling of the emulsion below the crystallization temperature helps in formation of the SLN dispersions. Note: (**A**,**B**) figures are adapted with permission from Ref. [23]. Copyright 2014, Elsevier, and (**C**) is adapted from Ref. [24].

Two methods of sonication are generally used depending on whether a probe tip ultrasonic disintegrator or a bath is used (Figure 3). While the bath sonicator is preferable for large volumes of diluted lipid dispersions, the probe sonicator is well suited for dispersions that require a large amount of energy in a low volume. Probe tip sonicators provide large energy to lipid dispersions; however, they can also induce lipid degradation due to overheating. Metal particles are often released by sonication tips into the dispersion, which should be removed by centrifugation before use. Bath sonicators are favored compared to probe tip sonicators for these reasons. The composition and concentration of lipids, duration, power, and temperature used for sonication all affect lipid dispersion particle size and size distribution [20].

The equipment used in this process is widely available at lab size, which is an advantage. This method, however, has drawbacks, such as a broader size distribution that extends into the micrometer range. Other disadvantages of this method include potential metal contamination and physical instability, such as particle growth when stored [21]. To control the size of nanoparticles, the frequency and strength of ultrasonication can be adjusted. Various research groups have attempted to prepare a robust solution by combining high-speed stirring with ultrasonication procedures carried out at elevated temperatures [22].

The probe ultrasonication technique was used by Bose et al. to produce quercetin SLN [25]. V. Venkateswarlu and K. Manjunath developed clozapine-loaded SLNs using hot homogenization followed by ultrasonication at a temperature greater than the melting point of lipids, and the results indicated that more than 90% of the drug was entrapped in SLN [26].

It is essential to investigate the SLN stability under various conditions, however several researchers ignore this important aspect. In a detailed investigation, DA Campos and coworkers studied phenolic compound rosmarinic acid (RA)-encapsulated SLNs prepared by the hot melt ultrasonication method. It was observed that the liquid state SLNs were stable for 90 days and the freeze-dried SLNs were stable up to 1 year [27]. These studies indicate that SLNs are suitable for herbal medicine delivery [28,29].

### 2.2. Solvent Emulsification Evaporation

Solvent emulsification evaporation is a dispersion technique to produce SLNs, which is a suitable method for thermolabile drugs such as ritonavir, chloramphenicol, and cyclopentolate. Here, the lipophilic drug and the lipid are dissolved in an organic solvent and thoroughly mixed to produce a homogeneous transparent lipid solution that is immiscible in water. Once the organic phase is prepared, it is emulsified with the appropriate amount of water (aqueous phase) using a high-speed homogenizer, giving us a coarse emulsion (o/w emulsion). This o/w emulsion is converted into a nanoemulsion with the use of a high-pressure homogenizer that breaks down globules into particles. To extract and eliminate the remnants of organic solvent, the nanoemulsion was kept in a hood or on a magnetic stirrer overnight with constant stirring. After the organic solvent evaporates, the lipid content precipitates in the bath, forming nanodispersion. Filtration by sintered disc filter funnels separates the precipitation of lipids in aqueous media. This method produces nanoparticles that are nonflocculated (single entity) with good entrapment quality [30]. Now, factors such as the type and amount of lipid, surfactant, and cosurfactant present in the organic phase are crucial in determining the particle size of the nanoparticles. For example, if we use a lipid content up to 5% by weight, it produces particles of size 30–100 nm. However, as the lipid content increases, the particle size also increases, perhaps due to the high viscosity of the dispersed phase causing a drop in homogenization efficiency [22]. This method produces nanoparticles that are compact and have a high encapsulation performance. The procedure can be optimized and expanded to produce large volumes of nanoparticles [31].

### 2.3. Solvent Emulsification-Diffusion

This is a modified and better version of the previously mentioned “solvent emulsification evaporation” process. Unlike the process described above, the solvent used in the solvent emulsification diffusion procedure is partly miscible with water, has lower toxicity and can be executed in both an aqueous and an oil form (for example, benzyl alcohol, butyl lactate, isopropyl acetate, methyl acetate, isovaleric acid and tetrahydrofuran). Thermodynamic equilibrium is maintained by saturating organic solvents with water. The basic mechanism of this technique is lipid crystallization caused by solvent migration from the internal organic phase to the external aqueous phase [31].

Nanoparticles with particle sizes of 100 nm and below can be obtained by this technique, in which surfactants play a vital role in optimizing the size. Similar to the previously described process, a drug dissolved in the organic solvent immediately precipitates out due to the diffusion of the organic solvent [22]. The lipid and drug are dissolved in a water-saturated solvent (internal phase) and emulsified using a mechanical stirrer with the dispersed phase (aqueous solution containing stabilizer). Water is added to the system after the o/w emulsion is formed to facilitate solvent diffusion into the continuous process, resulting in lipid precipitation in nanoparticulate form. This approach is effective and adaptable, has easy implementation and scaling-up properties, low physical tension (i.e., short exposure to elevated temperatures and mechanical dispersion) and eliminates the requirement of dissolving the drug in the melting lipid. The necessity to purify and concentrate the SLN dispersion and drug permeation into the aqueous process occurs quickly, resulting in low drug entrapment in SLN, which are both disadvantages of this approach [20].

### 2.4. Membrane Contactor

Due to its excellent scaling-up ability, the membrane contact technique is highly suitable for producing lipid nanoparticles on a large scale. It uses a simple apparatus to prepare solid lipid nanoparticles, and if the conditions are made favorable by careful selection of process parameters such as temperature or pressure, the particle size can be controlled, which makes the process more advantageous [22]. In this process, we have a lipid phase containing drug and an aqueous phase containing surfactant. The lipid phase is melted beyond its melting point and permeated through a porous membrane under pressure, which allows the formation of nanosized droplets. The aqueous phase keeps flowing tangentially in the internal membrane module, which sweeps away particles formed at the pore outlet of the membrane. The aqueous phase is retained at the lipid melting temperature. The formation of SLNs takes place when this preparation is solidified by cooling to room temperature or by keeping this preparation in a thermostatic bath of the desired temperature [31]. The particle size of lipid nanoparticles is influenced by many criteria, such as lipid phase temperature, pressure, aqueous phase temperature and cross-flow velocity, and membrane pore size [32].

Charcosset and coworkers experimented to learn the effects of different process parameters on the particle size of lipid nanoparticles where vitamin E-loaded SLNs were prepared using the membrane contractor technique [33]. They observed that there was an increase in lipid phase flux with a rise in the lipid phase pressure, so at the highest pressure, there was a slight decrease in particle size. Another interesting observation they found was that below the temperature of the lipid fusion point, the flux time increased, but smaller particles were formed. However, above the fusion point, the flux time decreased, and the particle size increased [5].

### 2.5. Double Emulsification

**Figure 4:**
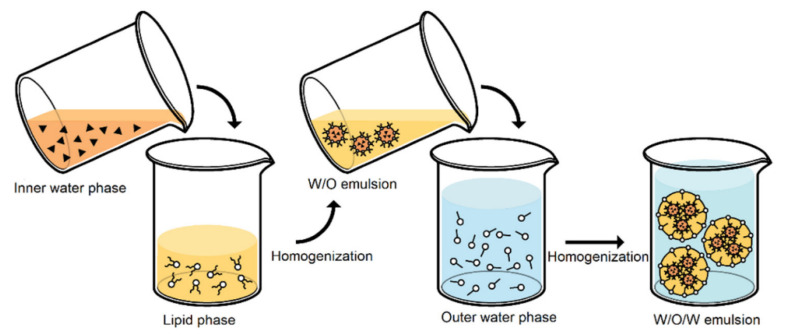
W/O/W double emulsion technique for the preparation of solid lipid nanoparticles. Reprinted from Ref. [35].

For the preparation of SLNs, APIs (active pharmaceutical ingredients) and hydrophilic proteins and peptides are acceptable [34]. A W/O emulsion is created in this process by mixing an aqueous solution consisting of medication with a mixture of melted oily phases at a temperature just above the melting point to obtain a clear solution. Excipients are used to stabilize the primary W/O emulsion (Figure 4), which is dispersed to the aqueous phase, cosurfactant, and surfactant to obtain a clear double W/O/W emulsion system. The warm double emulsion is then dispersed with cold and rinsed with a dispersion medium, resulting in the creation of SLNs [31].

Some of the disadvantages of the double emulsion method for SLN preparation are instabilities due to the coalescence of aqueous droplets inside the oily phase, rupturing of the coating on the surface of the interior droplets, and agglomeration of the oil droplets [36]. Trehalose is a disaccharide and is used as the most effective cryoprotectant in drying (specifically in the case of freeze-drying). This favors the preservation of the colloidal particle size of SLN formulations after reconstitution. For SLN preparations, a concentration of 1% SLN in trehalose (in water) or 20% trehalose (in ethanol-water mixture) yields the desirable results after drying of SLN.

### 2.6. Supercritical Fluid Extraction

**Figure 5:**
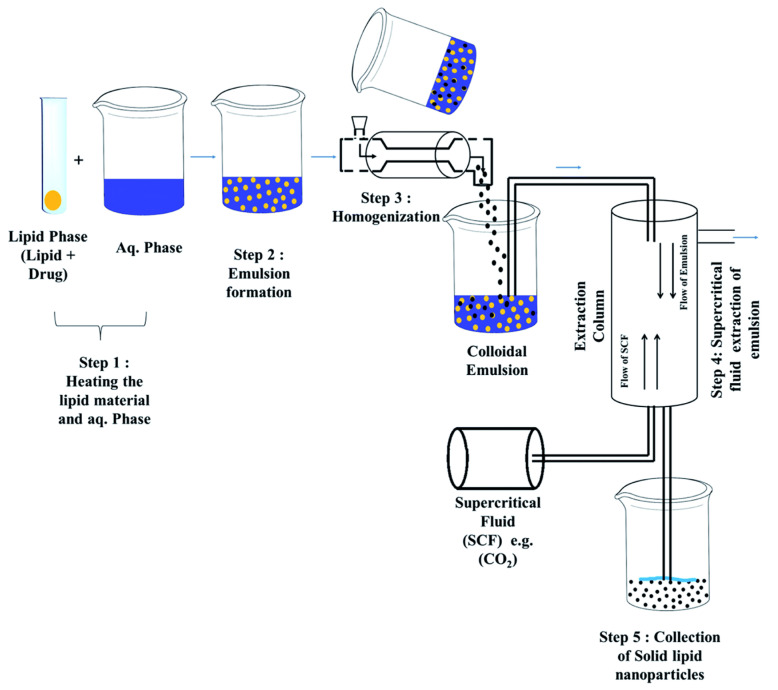
Supercritical fluid technique for the preparation of solid lipid nanoparticles. Reprinted from Ref. [17].

This is one of the most promising methods for producing solid nanoparticles, which works on the basic principle that lipid nanoparticles are formed from o/w emulsions by supercritical fluid extraction (SCF) [9]. The main advantage of this approach over other techniques is that it uses low temperatures (35 °C) and does not use organic solvents to make nanoparticles, i.e., solventless processing [37]. Carbon dioxide (CO_2_) is often used as SCF with or without the addition of other solvents (Figure 5). The particles obtained from this process have smaller particle sizes and distributions, have smooth surfaces and are free flowing, which justifies the advantages of this method. However, there are drawbacks to this approach, such as the cost, CO_2_’s low solvent strength, and the need for large volumes of CO_2_. SCF can be used as a solvent, swelling and plasticizing agent, antisolvent, or solvent for polymerization in dispersed media in nanoparticle processing [22]. The rapid expansion of supercritical CO_2_ solutions will generate SLNs [22]. CO_2_ with a purity of 99.99% is an excellent solvent for preparing SLNs using this form [32]. Gas-saturated solutions (GSS), such as ammonia, chlorodifluoromethane (CHClF_2_), 1,1,1,2-tetrafluoroethane (CH_2_FCF_3_) and ethane, are the best SCF [20] and aid in the melting of lipid materials, which then dissolve in the SCF under pressure with the lipid melt and GSS [38]. Spraying the saturated solution via the atomizer or nozzle causes it to expand and quickly release SCF, leaving fine dry lipid particles behind [30].

**Figure 6:**
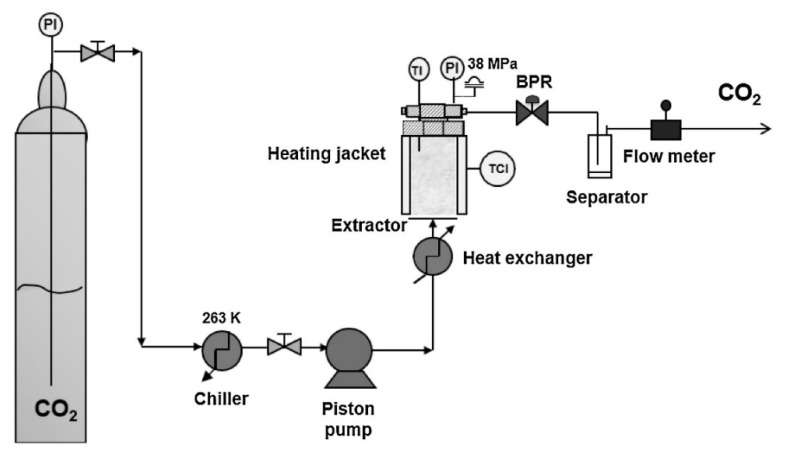
Scheme of the equipment used for supercritical fluid extraction of emulsions (SFEE). Reprinted with permission from Ref. [39]. Copyright 2016, Elsevier.

“Supercritical fluid extraction of emulsions (SFEE)” is the term used to describe the method of creating lipid nanoparticles using emulsions through SCF technology (Figure 6). The lipid component and the drug are dissolved in an appropriate surfactant-containing organic solvent, such as chloroform, to produce the organic phase. A high-pressure homogenizer is used to combine the organic solution with an aqueous solution that may additionally have a cosurfactant to create an o/w emulsion. The supercritical fluid (kept at fixed temperature and pressure) is currently supplied counter, while the o/w emulsion is delivered from one endpoint of the extraction unit (typically the top) at a fixed flow rate. Continuous solvent extraction from o/w emulsions is being used to form lipid nanoparticle dispersions [31].

### 2.7. Spray Drying Method

**Figure 7:**
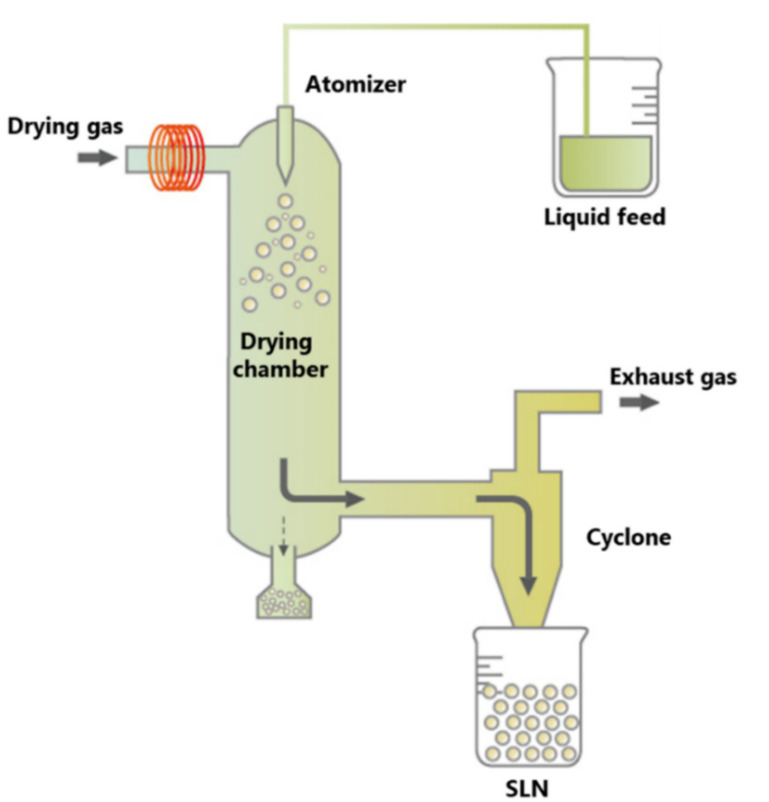
The spray-drying technique procedure is reproduced from [43].

Spray drying is the method used to create solid preparations from solutions and suspensions. The conversion of lipid nanoparticles from aqueous dispersion into a dry powder is very helpful for enhancing stability. Spray drying can change aqueous dispersions into a dry, fine, reconstituted powder that can be kept for a prolonged period [40]. Spray-drying is commonly used in the pharmaceutical industry. Spray drying is a one-step procedure for converting liquid feed to a dried atomized state (Figure 7). The feed is usually in the form of a solution; however, it can range from coarse to fine suspensions. The feed is first converted into spray form via different atomization methods, such as centrifugal, ultrasonic, or electrostatic atomization, which then is instantly put in contact with thermal hot gas, which leads to rapid solvent evaporation into a dried solid form. A cyclone separator, an electrostatic precipitator, separates hot air from dried solid particles. The capacity to control process parameters is the primary benefit of spray drying and it has specializations to manipulate a variety of parameters, such as feed composition, temperature, relative humidity, drying rate, and gas flow rate. As a result, spray drying technology permits the adjustment of particle characteristics, namely, size, size distribution, shape, density, and morphology, as well as macroscopic powder qualities, such as bulk density, tap density, powder flowability, and dispersibility. Physicochemical instability, which includes particle growth, unanticipated gelation, drug expulsion upon storage, or unexpected dynamic polymorphic changes of the lipid particles, is the main drawback of SLNs [41,42].

## 3. Potential Scale-Up Methods for SLN

SLNs are inexpensive to produce, have great physicochemical stability and are sterilized, lyophilized and scalable. These characteristics make SLNs attractive for manufacture on a large scale. However, scaling up creates challenges in many pharmaceutical operations. However, regarding the actual homogenization and the desired particle size distributions, HPH presents comparatively minimal issues [44]. This section describes various SLN scale-up methods.

### 3.1. High-Pressure Homogenization

The high-pressure homogenization technique (HPH) stands out among lipid nanoparticle (LNP) production techniques because of its ease of scale-up, lack of organic solvents, and lower production times. High-pressure homogenizers are commonly employed in a variety of industries, particularly the pharmaceutical industry, where they are used to make emulsions for parenteral nutrition. As a result, there are no regulatory issues with producing LNPs using this method, which is the most industrially possible. Common challenges faced in LNP production procedures, including HPH, are drug degradation brought on by the manufacture, lipid crystallization, gelation phenomena, supercooled melts, lipid and particle shape changes and the coexistence of various colloidal species. Nonetheless, these constraints can be controlled by closely examining production circumstances (temperature range, shear stress, and light) and enhancing drug carrier, formulation, and drug loading method selection [4]. The dispersion is made homogeneous at elevated pressure (500–2000 bar) through a narrow channel (a few micrometers) rapidly accelerated at high velocity (100 km/h) across a short distance in the HPH technique. Submicron particles are formed by high shear stress and cavitation forces [45].

For clinical batch/large batch manufacturing, the product container LAB 60 homogenization unit consists of two product containers (PC—product containers, H—homogenization block, and T—double-walled tubes). All connecting pipes are double-walled, and both connecting pipes and the homogenization head are temperature-controlled, allowing for finer control of the process parameters. In this manufacturing facility, a dissolver disc is used to prepare the presuspension (drug nanocrystals) as well as preemulsion (SLN, NLC, LDC) inside the first product container. The dispersion is transferred from this container back to the first one for the subsequent homogenizer runs. Steam can be used to sterilize the unit, and manufacturing can be conducted in an aseptic environment in a laminar airflow (LAF) unit. Rannie 118 can be used if larger dispersion volumes are necessary. At the highest production pressure of 1500 bar, this machine can homogenize 1.2 tons of material per hour. It is preferable to install two or three homogenizers in sequence rather than performing 10 or 20 runs by one homogenizer, especially regarding suspensions of drug nanocrystals. Because the homogenizers used are readily available and inexpensive, connecting three homogenizers in the sequence is cost-effective and cuts the homogenization/production time to one-third. Other types of piston-gap homogenizers can, of course, be utilized; nevertheless, the Avestin products are recommended (e.g., C55 and C1000). The potential contamination in the final product by using this equipment has proven to be exceptionally low, typically less than 1 ppm product contamination even under extremely difficult operational conditions, for example, 1500 bar, 20 homogenization cycles, and very hard crystalline materials in the case of drug nanocrystals [1,11,41].

The medium-scale production (40–50 mL) of SLN by HPH was demonstrated by Jenning et al. There were several types of homogenizers due to varying batch sizes, but the same functional rules were retained, and the changes from 40–50 mL were not significant. The study results revealed that a higher batch size had an impact on the product quality concerning particle size distribution and physical storage capacity. This might be attributed to machine performance by two distinct suppliers; however, it is established that it has been reproduced with batch-to-batch consistency. Moreover, this study revealed the importance of pressure and temperature for the hot homogenization technique and cooling step of the final product as major factors affecting the quality of the product. Excessive rapid cooling deteriorated the quality of the product, as cooling with water at 18 °C proved to be the ideal cooling condition [46].

R. Shegokar et al. scaled up the synthesis of stavudine-loaded SLN for intravenous injection on lab scale (40 g), medium scale (10 kg) and large scale (20/60 kg). The SLNs were made by homogenizing stavudine lipid melt distributed in a heated surfactant solution (preemulsion) under high pressure (800 bar). The APV Gaulin products LAB 40, LAB 60, Gaulin 5.5, and Avestin C50 piston-gap homogenizers were utilized both in continuous (circulation) and discontinuous modes [47].

In 2016, a study was published that focused on the development of a large-scale modular production line using coenzyme Q10-loaded NLCs and their continuous and scalable emulsification and homogenization mechanism. At a throughput of 25 kg/h (for lipid solution at a flow rate of 0.4 kg/min), the production line displayed excellent control over the emulsification and homogenization process, enabling particle sizes of NLCs to be under 210 nm. The author noted that the preemulsification temperature, homogenization pressure, and the number of homogenization cycles or passes were the primary parameters determining the properties of the final NLC product. Both batches were quite stable at room temperature (laboratory and large scale). The production line enabled digitalized networking of device elements and flexible characteristics for quick and affordable nanoparticle production [48]. The hot homogenization technique and the cold homogenization technique are the two main processing methods for SLN. The substance is dissolved or solubilized in the lipid, which is melted at approximately 5 ± 10 °C above its melting point in both techniques [7].

#### 3.1.1. Hot Homogenization

**Figure 8:**
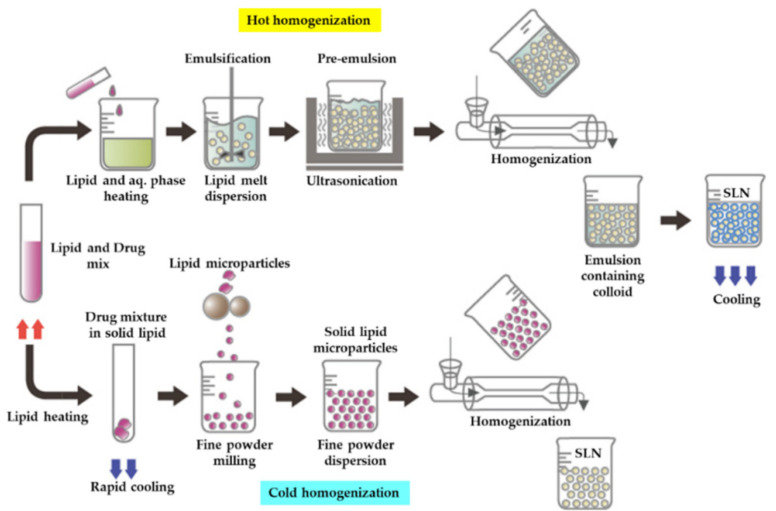
Homogenization technique: hot homogenization technique and cold homogenization technique. Reprinted from Ref. [43].

In particular, high temperature causes hot homogenization, which reduces particle size due to reduced inner phase viscosity, which is often ideal for medications with temperature sensitivity to a certain level when the material is exposed to an elevated temperature for such a limited time. Because of the limited particle size and inclusion of an emulsifier, the rate of drug and carrier degradation increases as the temperature rises (Figure 8). Lipid crystallization can be significantly slowed, and the sample can exist in a supercooled melt state for a few months. Since the drug is partitioned into the aqueous process during homogenization and many of the drug particles stay at the outermost surface of the SLNs when cooled, HHT is a poor technique for hydrophilic drug candidates, resulting in burst release [5]. The drug-containing melt is dispersed in a heated aqueous surfactant solution of the same temperature using the hot homogenization method. After homogenizing the collected preemulsion with a piston-gap homogenizer (e.g., Micron LAB40), cooling the hot O/W nanoemulsion to room temperature is performed, and the lipid recrystallizes, resulting in stable lipid nanoparticles [7].

A. Dingler and S. Gohla developed innovative and simple manufacturing and scaling-up techniques using hot HPH to prepare free drug and drug-incorporated SLNs at a substantial level. A piston-gap homogenizer, Micron LAB 40, was used for lab-scale production of SLN. The batch size ranged between 20 ± 40 mL, with the process being discontinuous. Furthermore, 2 ± 10 kg of SLN was produced by continuous or discontinuous HPH in a modified Lab 60 machine. The continuous production mode is advanced in producing SLN in 2 kg batches compared to the discontinuous mode due to a high dead volume of 0.5 mL. Fifty-kilogram batches were also tested in the study. With the existing production line and cetyl palmitate as a lipid matrix, 20 kg of SLN was prepared. Two hundred bars at homogenizer 1 (Gaulin 5.5) along with 500 bars at homogenizer 2 (Lab 60) were used to generate the first scaling-up batch. The second batch, which was formed with 500 bars at homogenizer 1 and 200 bars at homogenizer 2, examined the particle size. A homogenization pressure of 500 bar was used at each homogenizer for the third experiment. The authors suggested that using two homogenizers in the sequence is a great way to produce larger batches, and the particle size greatly depends on the total homogenization pressure used during the manufacture [49].

#### 3.1.2. Cold Homogenization

The cold homogenization method was introduced to address the major disadvantages of the hot homogenization technique, namely, drug degradation caused by temperature, drug distribution into an aqueous medium and homogenization, the complexity of the nanoemulsion crystallization step resulting in multiple changes, and/or supercooled melts (Figure 8) [3]. CHT prevents or reduces lipid melting, limiting the degradation of hydrophilic drugs in the aqueous process [5]. Thermal degradation is an instability issue for many formulations [50]. The method followed in cold HPH is that first the drug will be dissolved in molten lipids, and then the blend will be quickly cooled in liquid nitrogen or ice. Homogenous dispersions of drugs within the lipid matrix are made possible by this rapid cooling rate. The lipid–drug combinations are subsequently ground to a PS of 50–100 µm in a ball mill or a mortar. Lipid microparticles are suspended in surfactants containing cold aqueous solutions, which are further homogenized at the cold temperature (e.g., 0–4 °C) typically over 5–10 cycles at 500 bar [51].

### 3.2. Hot Melt Extrusion Coupled with HPH

**Figure 9:**
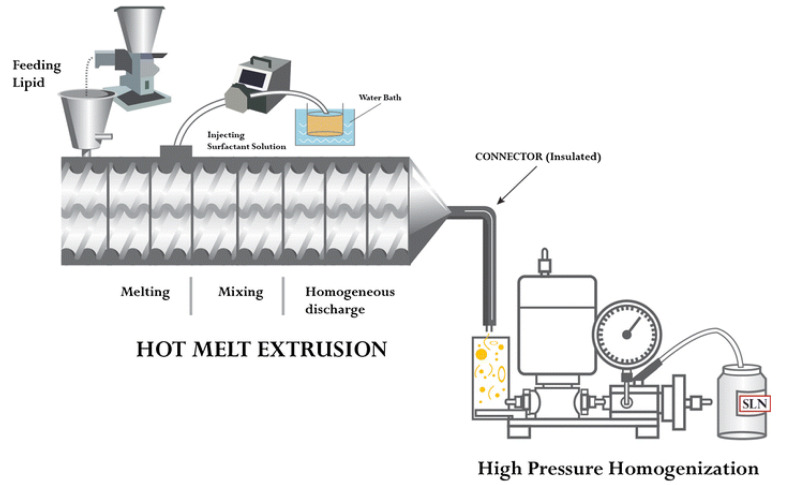
Schematic representation of continuous SLN synthesis using HME-connected HPH. Reproduced with permission from Ref. [54]. Copyright 2014, Elsevier.

The hot melt extrusion (HME) method is a continuous process of producing SLNs at higher temperatures and pressures to obtain products with uniform shapes, densities and morphologies [52,53]. The formulation of SLNs in the pharmaceutical industry can be produced by the combination of two methods: hot melt extrusion for the formation of SLNs and HPH to reduce particle size. By combining these two processes, one could produce a scalable process for SLNs by pumping the raw materials into the extruder barrel at an elevated temperature beyond the melting point of the lipids used and furthermore decreasing the size of the SLNs by connecting a high-pressure homogenizer at the end of a hot melt extruder barrel with an insulated connector. The abovementioned process demonstrated better size reduction and process parameters than the conventional process to produce SLNs. Among the investigated process parameters, the concentration of lipids, screw design and residence time play the most roles in impacting the size of the SLN. Patil et al. showed that by varying the abovementioned process parameters, SLNs less than 200 nm could be produced for a 60 mg/mL lipid solution at a flow rate of 100 mL/min. Process parameters, such as the liquid addition zone (ZL), barrel temperature zone (BT), speed of the screw extruder (SS), temperature of the liquid, design of the screw extruder, and lipid concentration, were improved for SLN formation by this HME-HPH combined method (Figure 9). As mentioned above, ZL, BT, and SS are critical in ensuring that the raw materials are melted and that the drug particles delivered are liquified in the lipid before exposure to the emulsifier solution. In addition, the HME temperature of the barrel zone for all zones, as well as the speed of the screw extruder, should be sufficient to melt the medication fully in the lipid solution, and when it comes in contact with the surfactant, the two phases combine to create an emulsion due to significant shear generation in the space between the extruder [54].

### 3.3. Liquid Flow-Focusing and Gas Displacing Method in Microchannels

**Figure 10:**
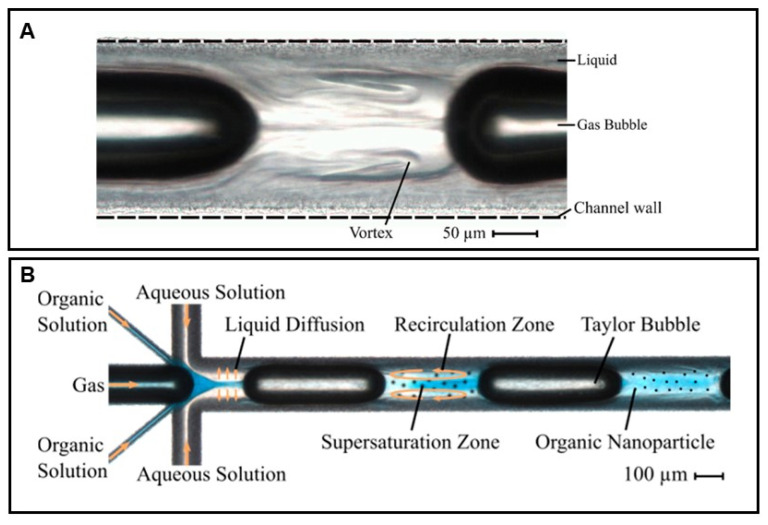
A microscopic view of a transparent microchannel showing vortices in the segmented gas-liquid flow (**A**) and microfluidic system with initial flow-focusing and subsequent Taylor flow (**B**) Reprinted from Ref. [58].

When compared to traditional approaches, solid lipid nanoparticles are an alternate medication delivery technology, and they have attracted a great deal of interest in research because of their applications in pharmaceutical areas, such as longer regulated release, long-term stability, and high tolerance. HPH, the microemulsion method, membrane contactor, ultrasonication, and supercritical fluid technology are some of the traditional methods for producing SLNs. However, these processes have some important operational parameters. Under overcritical conditions, such as greater temperature, speed, pressure, and suitable solvents, SLNs with narrow size distributions, small diameters, and lower zeta potentials are challenging to address. Microchannels (Figure 10), on the other hand, have been effectively employed to form microsized liquid droplets, nanosized particles, lipid microspheres, and nanoscale phospholipids [55,56,57].

One of the continuous and scalable approaches for producing solid lipid nanoparticles in microchannels is the liquid flow-focusing and gas displacement method (LFGDM). Microchannels are channels that have a cross-junction for both lipids and aqueous solutions, as well as a T-shaped junction for gas insertion. The creation of a lipid solution using a water-based organic solvent and surfactant concurrently along the cross-junction into the mainstream is used to focus liquid flow. Gas displacement is achieved by injecting an inert gas into the microchannel main flow upward to create a slug flow of the gas-liquid mixture through the T-shaped junction. The liquid flow-focusing method can form SLNs of small diameters and narrow size distributions, according to the idea behind the LFGDM method, which is based on hydrodynamic focusing (LFM) [57]. Producing SLNs in microchannels by LFM is a complex process that involves integrating liquid phase flow-focusing, solvent mass transfer, and the flow of a suspension mixture with nanosized particles (Figure 10). However, the LFM method has a blockage problem of particles inside the microchannels, as they may disintegrate in the continuous method. Therefore, to overcome this problem, gas sparging continuation along with LFM using gas-liquid slug flow could be used to reduce fouling or deposition of solids inside the channels. The LFGDM rationale consists of two important steps: The first is the formation of lipid and aqueous solution streams through the liquid flow-focusing process, and the second is the gas insertion procedure, which allows the gas-liquid slug suspension of SLNs to travel via microchannels in a smooth and unbroken manner. The lipid solution is injected into the mainstream simultaneously with the aqueous solution through two distinct branch channels during the first step. At the cross section of the main channel, these two streams meet in such a way that the lipid solution flow tends to occupy the central region of the main channel, surrounded by the aqueous phase. Supersaturation of the lipid occurs after diffusion of the organic solvent with the lipid solution into the aqueous phase, resulting in the formation of SLNs. Following the creation of SLNs, gas bubbles larger than the diameter of the microchannels split the upper liquid streams. They result in the formation of Taylor bubbles and the suspension of gas-liquid SLNs. The flow pattern is caused by the production of slug suspension and tiny bubbles surrounded by a thin liquid sheet. Because of these Taylor bubbles and liquid slug flow motions, the deposition of SLNs into the microchannels is prevented, ensuring the smooth and free passage of SLNs via the microchannels and achieving continuous SLN production [59,60,61].

### 3.4. Nanoprecipitation Using Static Mixers

Nanoprecipitation is a simple and quick method for producing SLNs with better scale-up potential. This method involves dissolving the lipid with organic solvents (e.g., ethanol, acetone, etc.) and adding it to water at the same time, causing supersaturation of solid lipids in the lipid–water mixture, resulting in solid lipid nanoparticle precipitation under proper and practical conditions. Polymeric nanomedicines have been extensively explored by the nanoprecipitation technique [62,63]. Nanomedicine is a broadly applied term for formulations such as liposomes [64], polymersomes [65,66], polymeric nanoparticles [67], inorganic nanocarriers [68], nanofibers [69,70] protein nanoparticles [71], nanosuspensions [72], and emulsions [73]. Using this technique, one can obtain smaller and more homogenous particles, so the selection of the solvent needs to have the attributes to diffuse into the water in less time and homogeneously [74].

Mixing is an important aspect, as it should be completed before precipitation. For large-scale production of SLNs, conventional tanks are not preferred because they are slow, and mixing is nonuniform. Previously, several mixing devices, such as microfluidics impinging mixer jets and T-mixers, have been reported to ensemble fast and rapid homogenous mixing in the nanoprecipitation process [75]. Previous studies have shown that microchannel mixers have been explored for the synthesis of SLN through nanoprecipitation techniques, but the yield of productivity was low [76]. Static mixers were chosen because they eliminate the issues mentioned above and provide efficient mixing for SLN manufacturing on a wide scale. Static mixers are composed of similar and almost motionless parts that have tortuous structures and are in the tube, column, or reactor. In addition, there are many advantages of static mixers, such as operational cost, less space requirement, energy requirement and continuous operation. Dong et al. [14] demonstrated that process variables such as the amount of mixing materials, lipid concentration, and flow rate have a significant impact on the size reduction of SLNs. Table 1 summarizes the scale-up methods of SLNs.

### 3.5. Microemulsion-Based Method

In this technique, lipid nanoparticles are made using microemulsification. This technique is highly reliable and efficient since it does not require energy to produce nanoparticles, making it ideal for incorporating thermolabile drugs into lipid nanoparticles. Composition, pH, and temperature all have a significant impact on product quality. Lipid nanoparticles are made from a heated microemulsion that contains low melting lipids, an emulsifier, a cosurfactant, and water. This microemulsion is stirred into surplus cold water, causing the lipid phase to precipitate, creating nanoparticles, i.e., dispersion of warm o/w (oil-in-water) microemulsion in cold water [22]. The excess water is then removed using an appropriate technique, such as ultrafiltration or lyophilization. This technique has the disadvantage of requiring the removal of excess water from lipid nanoparticles as well as a high concentration of surfactant and cosurfactant. This leads to the creation of a very diluted SLN dispersion, which necessitates further processes such as diafiltration or lyophilization to create a stable concentrated product, which must then be reconstituted for compliance use. In another study, researchers performed microemulsification to prepare, characterize and scale-up sesamol-incorporated solid lipid nanoparticles. They developed scaled-up samples of 1 L to 100 L to assess the market potential of the nanoparticles and to characterize the SLN formulation with different in vitro characteristics, such as particle size, percentage entrapment efficiency (EE), transmission electron microscopy, infrared spectroscopy, differential scanning calorimetry, powder X-ray diffraction studies and in vitro release. On a lab scale, they succeeded in scaling up the 100× batch [77]. Challenges in scale-up methods are summarized in Table 2.

## 4. Conclusions and Future Perspectives

In conclusion, SLNs appear to be an appropriate delivery system for drugs that have poor solubility in water, lower chemical stability, and a short half-life. Furthermore, the studies suggest that by modifying formulation processing and hardware aspects, it is possible to prepare lipid nanoparticles with a constant and repeatable quality of production. There are many production challenges such as polymorphism, phase separation and sterilization resulting from processing steps. Polymorphism can be addressed by using temperature-controlled methods such as SCF, however there are no attempts on the reported literature for scale-up of SLNs using SCF. An optimized lyophilization process is essential in dealing with phase separation. It is often reported that the solid particles tend to be more stable and decrease the microbial growth incidents drastically. One bottleneck is the sterilization process because thermolabile substances and lipids are sensitive to gamma irradiation and steam methods. This problem can be easily solved by using filtration methods but the nanoparticle batch capacity for such methods is often in the range of 2–5 L; moreover, the filter pore size is the deciding factor in screening. The protein corona formation and enzyme degradation are another obstacle in translation, however the recent advances in the bio corona characterization estimation methods and high throughput screening (HTS) methods have made it possible to study this before moving forward to the clinical stage.

To fully benefit from the diverse uses of SLN formulation, research institutes and industries should encourage their scale-up efforts to bring them onto the market. Like nanosuspensions, lipid nanoparticles are capable of being lyophilized and spray dried in a subsequent stage of processing. All other commercial standards, such as a clean room, a separate manufacturing facility, machine validation, flooring, safety, and employee training, still apply to lipid nanoparticle products. In recent years, liposome regulatory guidelines have been clearly defined by authorities, which may provide a broad understanding of SLNs. Furthermore, ongoing clinical studies indicate that these technologies will eventually reach the pharmaceutical market in the coming years.

## Figures and Tables

**Table 1:** Summary of the scale-up methods of solid lipid nanoparticles.

Sr. No.	Title of the Publication	Methods	Conditions	Results	Pros, Cons, and Remarks
1	Synthesis and stability of stavudine solid lipid nanoparticles ranging from lab to industrial-scale [47]	HPH **(Text reference: Section 3.1)**	▪ **Drug:** Stavudine ▪ **Pressure:** 500 to 30,000 psi/3.5–207 MPa/35–2000 bar ▪ **Rpm:** 220 ▪ **Cycles:** 5 ▪ **Flow rate:** 15 L/h ▪ **Temperature:** 80 °C	▪ **Particle size:** 50–100 nm ▪ **PDI:** 0.150 ▪ **Zeta potential:** −20 to −24 mV	▪ **Pros:** Widespread and easy to handle ▪ **Cons:** High energy is needed, polydisperse, biomolecules get damaged ▪ **Remarks:** Particle size is proportional to pressure and the number of cycles
2	Continuous manufacturing of solid lipid nanoparticles by hot melt extrusion [54]	Hot melt extrusion followed by a high-pressure homogenizer **(Text reference: Section 3.2)**	▪ **Lipid phase:** 60 mg/mL ▪ **Flow rate:** 100 mL/min ▪ **Screw speed:** 240, 160 ▪ **Pressure:** 1000 bar ▪ **Barrel temp:** 150–100–83 °C (zone2–zone3–zone4)	▪ **Size:** 200 nm ▪ **PDI:** 0.264 ± 0.015 ▪ **Zeta potential:** −30.6 ± 0.15 mV	▪ **Pros:** No organic solvent needed, continuous manufacturing decreased space requirements, labor, and resources ▪ **Cons:** Special HME instrument needed for melting ▪ **Remarks:** Uniform shape and density
3	Continuous production of solid lipid nanoparticles by liquid flow-focusing and gas displacing method in microchannels [61]	The liquid flow-focusing and gas displacing methodin microchannels **(Text reference: Section 3.3)**	▪ **Drug:** Vitamin E ▪ **Flow velocity of lipid solution:** 0.0300 m/s ▪ **Surfactant concentration:** 0.5% Poloxamer ▪ **Temperature:** 27 °C	▪ **Size:** 50–280 nm	▪ **Pros:** Simple and devoid of overly complex processes such as rapid speed, toxicological solvents, and high pressure ▪ **Cons:** Deposited volume variability ▪ **Remarks:** Small sizes and confined range of diameters.
4	Solid lipid nanoparticles: Continuous and potential large-scale nanoprecipitation production in static mixers [14]	Nanoprecipitation (Static mixers) **(Text reference: Section 3.4)**	▪ **Drug:** Fenofibrate ▪ **Flow rate:** lipid solution: 50 mL/min, aqueous solution: 450 mL/min ▪ **Lipid concentration:** 25 mg/mL	▪ **Size:** 160.7 ± 1.5, 159.3 ± 2.0 and 165.7 ± 3.5 nm for the SLN precipitating from static mixers having 6,12 and 18 elements, respectively ▪ **PDI:** 5% drug: 0.249 ± 0.018, 10% drug: 0.201 ± 0.017, 15% drug: 0.177 ± 0.019 ▪ **Loading efficiency:** 5% (Drug): 31.37 ± 0.02%, 10 Drug): 15.15 ± 0.17%, Drug): 6.86 ± 0.28	▪ **Pros:** HPH and microemulsion methods have been examined for the feasibility of scaling up, HPH is a rather energy- and time-consuming procedure, while the microemulsion method needs a substantial amount of surfactants and nanoprecipitation is a quick, easy and facile process
5	Preparation, characterization, and scaling up of sesamol incorporated solid lipid nanoparticles [77]	Microemulsion-based method **(Text reference: Section 3.5)**	▪ **Drug:** Sesamol ▪ **Rpm:** 5000 rpm for 2 h ▪ **Surfactant:** soy lecithin (80 mL) ▪ **Water phases:** 500 mL ▪ **Temperature:** 80–85 °C	▪ **Particle size:** 106.6 nm ▪ **PDI:** 0.303 ▪ **Encapsulation Efficiency:** 72.57 ± 5.20% ▪ **In vitro release:** 90% of release in less than 16 h	▪ **Pros:** No energy is required ▪ **Cons:** Solvent which distributes rapidly into the aqueous phase (acetone) ▪ **Remarks:** Final product is dry powder instead of suspension

**Table 2:** Challenges in scale-up methods and stability issues [78].

Sl. No.	Method	Challenges	Remarks
1	Coacervation	Polymorphism	In this method, the use of fatty acids for LNP preparation can result in polymorphs due to the recrystallization of lipid matrices.
2	Hot homogenization	Polymorphism	The use of triglycerides in LNP production can result in polymorphs when its metastable α form transforms into a more stable β form upon storage, causing increases in melting point, drug leakage, and NP aggregation.
3	Spray drying and congealing process	Polymorphism	Rapid solvent evaporation led to unstable polymorphic forms.
4	Spray drying	Phase separation	Nanoparticles get aggregated in a reversible (flocculation) or irreversible (coalescence) fashion. Gelling phenomenon upon storage. Storage stability can be increased by converting LNP suspension into powder form by spray drying (coalescence can be prevented by adding carbohydrates) or lyophilization (aggregation can be avoided by the use of cryoprotectants).
5	Gamma irradiation	Sterilization	Chemical degradation of lipids because of irradiation.
6	Autoclaving	Sterilization	The temperature used in this method affects LNP stability.
7	Sterile filtration	Sterilization	Used solely for particles whose size range falls within that of filter pores.
8	Stability investigation in biological fluids	Gastrointestinal (GI) fluids and circulatory protein, serum albumin	Lipid matrix degraded by the enzymes present in GI fluids. Particle size increased because of protein adsorption on the NP surface

## Data Availability

The authors declare that the review article materials of this study are available within the article.

## Abbreviations

SLN	Solid Lipid Nanoparticles
GM	Glyceryl Monostearate
GB	Glyceryl Behenate
GP	Glyceryl Palmitate
MM	Myristyl Myristate
CP	Cetyl Palmitate
SCF	Supercritical Fluid Extraction
CO_2_	Carbon Dioxide
GSS	Gas Saturated Solutions
CHClF_2_	Chlorodifluoromethane
CH_2_FCF_3_	1,1,1,2-Tetrafluoroethane
SFEE	Supercritical Fluid Extraction of Emulsions
APIs	Active Pharmaceutical Ingredients
HPH	High Pressure Homogenization
LNP	Lipid Nanoparticle
PC	Product Containers
NLC	Nanostructured Lipid Carriers
LDC	Lipid Drug Conjugate
LAF	Laminar Air Flow
HHT	Hot Homogenization Technique
CCT	Cold Homogenization Technique
HME	Hot Melt Extrusion
PS	Particle Size
ZL	Liquid Addition Zone
BT	Barrel Temperature Zone
SS	Speed of Screw Extruder
LFGDM	Liquid Flow Focusing and Gas Displacement Method
EE	Entrapment Efficiency
EPR	Enhanced Permeation and Retention
CNS	Central Nervous System
BBB	Blood Brain Barrier
RES	Reticuloendothelial System
CLZ	Clotrimazole
ALA	Alphalipoic Acid
UV	Ultraviolet
